# Complementary crosstalk between palmitoylation and phosphorylation events in MTIP regulates its role during *Plasmodium falciparum* invasion

**DOI:** 10.3389/fcimb.2022.924424

**Published:** 2022-09-29

**Authors:** Zille Anam, Geeta Kumari, Soumyadeep Mukherjee, Devasahayam Arokia Balaya Rex, Shreeja Biswas, Preeti Maurya, Susendaran Ravikumar, Nutan Gupta, Akhilesh Kumar Kushawaha, Raj Kumar Sah, Ayushi Chaurasiya, Jhalak Singhal, Niharika Singh, Shikha Kaushik, T. S. Keshava Prasad, Soumya Pati, Anand Ranganathan, Shailja Singh

**Affiliations:** ^1^ Special Centre for Molecular Medicine, Jawaharlal Nehru University, New Delhi, India; ^2^ Department of Life Sciences, School of Natural Sciences, Shiv Nadar University, Greater Noida, Uttar Pradesh, India; ^3^ Center for Integrative Omics Data Science, Yenepoya (Deemed to be University), Mangalore, India; ^4^ Center for Systems Biology and Molecular Medicine, Yenepoya (Deemed to be University), Mangalore, India

**Keywords:** malaria, *plasmodium falciparum*, crosstalk, myosin A tail interacting protein (MTIP), post-translational modifications

## Abstract

Post-translational modifications (PTMs) including phosphorylation and palmitoylation have emerged as crucial biomolecular events that govern many cellular processes including functioning of motility- and invasion-associated proteins during *Plasmodium falciparum* invasion. However, no study has ever focused on understanding the possibility of a crosstalk between these two molecular events and its direct impact on preinvasion- and invasion-associated protein–protein interaction (PPI) network-based molecular machinery. Here, we used an integrated *in silico* analysis to enrich two different catalogues of proteins: (i) the first group defines the cumulative pool of phosphorylated and palmitoylated proteins, and (ii) the second group represents a common set of proteins predicted to have both phosphorylation and palmitoylation. Subsequent PPI analysis identified an important protein cluster comprising myosin A tail interacting protein (MTIP) as one of the hub proteins of the glideosome motor complex in *P. falciparum*, predicted to have dual modification with the possibility of a crosstalk between the same. Our findings suggested that blocking palmitoylation led to reduced phosphorylation and blocking phosphorylation led to abrogated palmitoylation of MTIP. As a result of the crosstalk between these biomolecular events, MTIP’s interaction with myosin A was found to be abrogated. Next, the crosstalk between phosphorylation and palmitoylation was confirmed at a global proteome level by click chemistry and the phenotypic effect of this crosstalk was observed *via* synergistic inhibition in *P. falciparum* invasion using checkerboard assay and isobologram method. Overall, our findings revealed, for the first time, an interdependence between two PTM types, their possible crosstalk, and its direct impact on MTIP-mediated invasion *via* glideosome assembly protein myosin A in *P. falciparum*. These insights can be exploited for futuristic drug discovery platforms targeting parasite molecular machinery for developing novel antimalarial therapeutics.

## Introduction

*Plasmodium falciparum (P. falciparum)* has remained a versatile and highly adaptable pathogen causing severe mortality in humans (Health Organization, 2019). The parasite successfully cycles through the non-vertebrate (female Anopheles mosquito) and vertebrate (human) hosts. The parasite enters its human hosts through the saliva upon a mosquito bite that injects sporozoites into blood. The sporozoites first reach the liver where the asexual reproduction happens; this increases the parasite numbers and leads to an eventual release of merozoites into the bloodstream leading to the infection of red blood cells (RBCs). The parasite is also conveniently able to undergo stage transformations, and reinfect, thereby causing drastic effects in humans. Notably, biomolecular events like post-translational modifications (PTMs), including phosphorylation, methylation, and ubiquitylation, are known to govern survival, life-cycle progression, biology, and pathogenesis in the Apicomplexan parasites by modulating the proteomic diversity (Jortzik et al., 2011; Douse et al., 2012; Doerig et al., 2015; Vembar et al., 2016; Yakubu et al., 2018).

**Figure 1:**
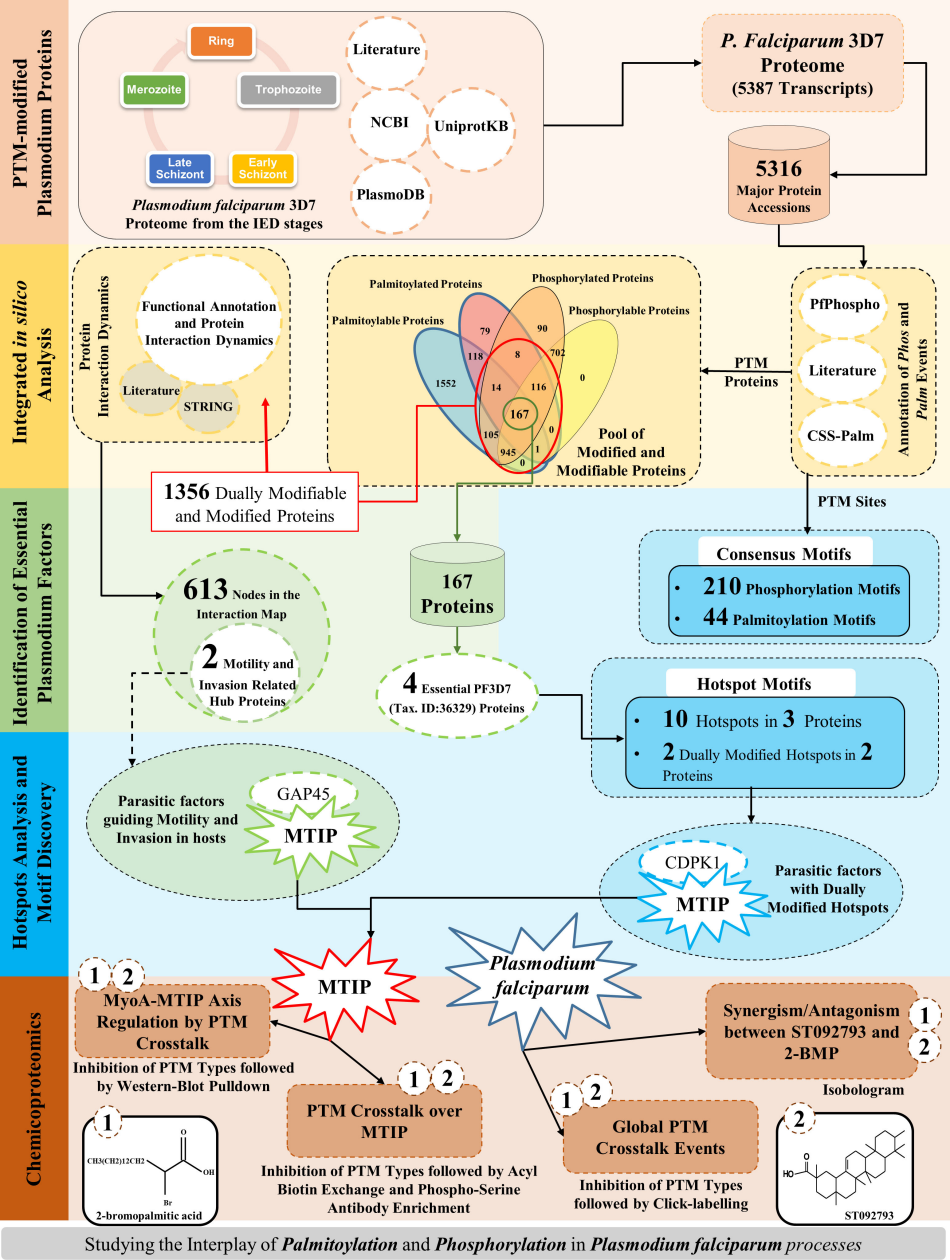
A graphical summary.

Annotation of PTM modifications involved in critical biomolecular processes is a well-established tool to identify parasite-specific molecular targets (Park et al., 2015; Yakubu et al., 2018). Phosphorylation and palmitoylation are the two most critical PTM types known in *P. falciparum* and have been shown to play crucial roles during various stages of the parasite life-cycle progression (Jones et al., 2012; Yakubu et al., 2018; Perrin et al., 2020). Existing studies have shown that among all the modified proteins, phosphorylated and palmitoylated proteins, mostly expressed on the parasite surface and the inner leaflet, are responsible for host cell invasion processes (Jones et al., 2012). The force necessary for the *Plasmodium* spp. parasites to glide and invade the host RBCs is generated by the glideosome complex. The differential states of the gliding and invasion machinery have been earlier associated with the phosphorylation and/or palmitoylation events in *Plasmodium* parasites (Douse et al., 2012; Jones et al., 2012; Ridzuan et al., 2012; Alam et al., 2015; Lasonder et al., 2015). However, there is a paucity in the understanding if these PTM types can crosstalk to regulate parasite processes during the asexual growth stages. Toward this, our study has shown for the first time 1) an experimental documentation of a cross-regulation of phosphorylation and palmitoylation at both global and individual protein levels and 2) interdependence of phosphorylation and palmitoylation over MTIP governing its essentialness, and interactions with myosin A during the asexual intraerythrocytic development of parasites. Additionally, a catalog of all PTM hotspots and specific proteins associated with parasite motility and invasion-related processes have been documented. The detailed strategy and findings of this study have been organized as a schematic representation (**Figure 1**).

## Materials and methods

### *In silico* prediction of phosphorylation and palmitoylation in *Plasmodium falciparum*

The updated proteome of *P. falciparum* 3D7 was indexed from databases like PlasmoDB Release 58 (Amos et al., 2022), National Center for Biotechnology Information or NCBI (GCA_000002765), Pf-Phospho (Gupta et al., 2022), and UniProtKB Release 2022_02 (Bateman et al., 2021), and also from other curated resources (Solyakov et al., 2011; Jones et al., 2012; Lasonder et al., 2012; Govindasamy et al., 2016; Kumar et al., 2017a; Kumar et al., 2017b; Pease et al., 2018; Blomqvist et al., 2020; Gupta et al., 2022). Probable pseudogenes were removed from the library, and only the genes with a protein coding potential were selected for downstream processing.

Phosphorylation events specific to the proteome assembly were retrieved from curated mass spectrometry resources (Ganter et al., 2017), databases (PlasmoDB, UniProtKB, PfPhospho, and NCBI), and Pf-Phospho (Gupta et al., 2022) prediction results. Proteome-specific palmitoylation events and sites were cataloged from CSS-Palm prediction results (Ren et al., 2008), publicly available mass spectrometry resources (Jones et al., 2012), and systematic database search results from PlasmoDB, NCBI, and UniProtKB. Consensus sequence windows around the central modification sites were then discovered for each of the modification types individually within all the modified proteins. The sequence windows were discovered and ranked based on their overall scores using the MoMo tool (Cheng et al., 2019) of the MEME Suite (Bailey et al., 2015).

All the proteins featuring in the union between parasite phosphoproteome and palmitome cataloged herein (curated or predicted) were termed as “Dually Modifiable” proteins. Interaction networks were generated between the dually modifiable proteins, with the highest confidence score (0.9 and above) and high FDR stringency (1% and below). The dually modifiable proteins mapped to the STRING-db were clustered into multiple functional groups using the k-mean clustering tool in the STRING database (Szklarczyk et al., 2021). The dually modifiable proteins which were predicted and referenced in the publicly available mass spectrometry datasets/curated databases (described in Section 3.1) were finally categorized as “Dually Modified” proteins. A high confidence list of essential parasitic factors with no paralogs in the human host proteome was prepared from the intersection subset (A∩B) of curated database searches and literature mining results. (Ali et al., 2021; Bateman et al., 2021; Amos et al., 2022). The dually modified proteins were subsequently mapped to the essential proteins. Only essential parasite factors exhibiting a strong probability of dual modifications were considered for the indexing of PTM sites and PTM hotspot discovery processes. For the hotspot analysis, all the modified residues were cataloged into motifs, each one featuring ±5 amino acids. Every motif (of 11 amino acids) exhibiting a localization of ≥3 modified residues including the central PTM site was defined as PTM hotspots. The PTM hotspots were then categorized based on whether they featured amino acid residues corresponding to a single PTM type or both PTM types. All the overlapping hotspots were collated as actual hotspot stretches. All R-programming codes were drafted in accordance with the available literature (Aggarwal et al., 2020). Suitable filters were applied to classify the proteins with dually modified PTM hotspots.

The functional annotation tasks were executed for the proteins and clusters using DOSE (Yu et al., 2015) and “enrichKEGG” function of the clusterProfiler package (Yu et al., 2012; Wu et al., 2021) in R programming. Other annotations were retrieved from the PlasmoDB, UniProtKB, and KEGG databases (Kanehisa and Goto, 2000; Kanehisa, 2019; Kanehisa et al., 2021). All KEGG annotations and pathway components were visualized using the web-based KEGG mapper and the Bioconductor (v3.15) package *Pathview* (Luo and Brouwer, 2013). All chemical compound structures were generated using the PubChem Sketcher Tool (Ihlenfeldt et al., 2009). Important sites on the proteins of interest were marked interactively using the freely available visualization tool Protter (Omasits et al., 2014).

### Anti-MTIP antibody generation

Purified recombinant MTIP protein residues 61-204 (XP_001350849) were resolved by SDS-PAGE and checked for purity. Approximately 100 μg of this was mixed with complete Freund’s adjuvant for the first dose and incomplete Freund’s adjuvant for the booster dose after 7 days. The mixture was administered to female BALB/c mice (6–8 weeks old) subcutaneously, and the bleed was collected after 7 days of the first booster. Pre-immune sera were collected before immunizing the mice. Mice were administered three booster doses, and sera were collected every 7th day of the injection. The specificity of the anti-MTIP antibody was detected by probing *P. falciparum* lysates with anti-MTIP.

### Acyl biotin exchange

The palmitoylated pool of proteins was purified using a modified version of the original acyl-biotin exchange (ABE) protocol described in Wan et al. (2007). Purified segmented schizonts (~40–42 h post-invasion) were treated with 10 µM ST092793 [originally identified from the virtual screening of MyriaScreen II Diversity Collection library that is composed of drug-like compounds in Jain et al. (2020)] and 50 µM 2-bromopalmitate (2-BMP) for 4 h each. The treated parasite pellet was resuspended in a lysis buffer (150 mM NaCl, 50 mM Tris–HCl, 5 mM EDTA, pH 7.4) containing 10 mM N-ethylmaleimide (NEM). NEM causes the irreversible blockage of unmodified cysteine thiol groups. The mixture was incubated at 4°C for 16 h. Detergent-sensitive and detergent-resistant fractions were then separated by centrifugation at 13,000 rpm for 30 min at 4°C. The detergent-sensitive fraction was then processed for ABE. Briefly, after centrifugation, the protein from supernatant fractions was precipitated using the methanol/chloroform precipitation method (methanol:chloroform:water, in ratio 3:1:2). Precipitated proteins were then solubilized in four volumes of solubilization buffer containing 4% SDS, 50 mM Tris, and 5 mM EDTA (pH 7.4) in the presence of 10 mM NEM and incubated overnight at 4°C. Again, the precipitation step was repeated to remove NEM and the precipitated protein elutes were solubilized in four volumes of solubilization buffer in the presence of hydroxylamine (HA) buffer (0.7 M HA, 0.2 mM HPDP-biotin, 50 mM Tris pH 7.4, 0.25 Triton X-100; in Milli-Q water) for 2 h at 37°C. The final precipitation step-coupled resolubilization was performed in HPDP-biotin buffer (0.2 mM HPDP-biotin, 50 mM Tris pH 7.4, 150 mM NaCl, 5 mM EDTA, 0.2% Triton X-100; in Milli-Q water) for 2 h. At this step, a small amount of fraction from each of the treated samples was aliquoted. This served as the loading control. Further, each of the treated biotinylated samples was then pulled down using streptavidin beads and elution of proteins was performed in the presence of elution buffer (0.1% SDS, 0.2% Triton X-100, 1% β-mercaptoethanol; in Milli-Q water). The palmitoylated elutes were observed by Western blotting using the mouse anti-MTIP antibody (1:10,000). For the loading control, a lysate fraction aliquoted before streptavidin pull-down was observed by Western blot using mouse anti-MTIP. Band intensities were calculated using ImageJ software and plotted as fold change in MTIP expression before and after streptavidin pull-down.

### Phospho-serine antibody-based pull-down assay

The pool of phosphorylated serine was pulled down from *P. falciparum* parasites treated with ST092793 and 2-BMP using Pierce Co-Immunoprecipitation Kit (Catalog Number 26149) as per manufacturer’s protocol using a phosphoserine antibody (Catalog Number sc-81514). The elutes were analyzed by Western blotting using mouse anti-MTIP antibody (1:10,000). An intensity graph was normalized with their respective inputs taken out before pull-down with phospho-serine antibody and plotted as bar graph.

### MyoA/MTIP interaction pull-down assay

The biotin-tagged MyoA tail was bound to streptavidin beads (Catalog Number 20347) as per the manufacturer’s instructions. Total cell lysates prepared in lysis buffer (150 mM NaCl, 50 mM Tris–HCl, 5 mM EDTA, pH 7.4) were allowed to interact with the streptavidin-bound MyoA tail overnight at 4°C (MyoA peptide residues 798–818 was synthesized from GenScript). The beads were washed with phosphate-buffered saline (PBS) followed by elution in 1× SDS loading dye. The elutes were analyzed by Western blotting using mouse anti-MTIP antibody (1:10,000). The intensity of each band was normalized with their respective input aliquoted before pull-down and plotted as a bar graph.

### Click chemistry

ODYA-palmitic acid (Alk-C16) (Invitrogen, USA) was added to the untreated set of *P. falciparum* 3D7-infected RBCs at a final concentration of 100 µM in PBS, for 4 h at 37°C with constant shaking. For 2-BMP, ST092793, and ST092793+2BMP treatment, cells were also simultaneously treated with 2-BMP (50 µM), ST092793 (10 μM), and 2-BMP+ST092793, respectively, for 4 h at 37°C. Following treatment, RBCs were pelleted down at 1,500 g. Thereafter, samples were washed thrice with ice-cold PBS, fixed with glutaraldehyde (0.25% in PBS) for 15 min at 4°C with shaking, and further permeabilized using 0.01% Triton X-100 (Sigma-Aldrich, USA) in PBS at room temperature for 5 min (shaking). After each step, a minimum of two washes with 1× PBS were done. These samples were then subjected to a click labeling reaction in 100 µl of dye mix (in PBS) containing 0.1 mM azide dye (Oregon Green^©^ 488, Thermo Fisher Scientific, USA), 1 mM tris-(2-carboxyethyl)-phosphine hydrochloride (TCEP, Sigma-Aldrich, USA), and 1 mM CuSO_4_ (Sigma-Aldrich, USA) in water for 1 h. After incubation, cells were pelleted down and washed twice with 1× PBS. For microscopy, a drop (3–5 µl) of the above sample was used to make a smear on a glass slide, mounted with Gold Antifade DAPI (Molecular Probes, USA), and analyzed using a Nikon A1 confocal microscope. NIS-Elements software was used for the processing of images. Mean fluorescent intensity (MFI) was determined for a single cell by measuring fluorescence intensity and plotted as a bar graph showing the MFI at a single-cell level. All imaging parameters were held constant during acquisitions.

### *Plasmodium falciparum* growth inhibition assay

In order to analyze the effect of the crosstalk on *P. falciparum* growth, purified segmented schizonts [~44–48 h post-invasion (hpi)] were purified and diluted to a final parasitemia 2% (two schizonts per 100 RBCs) and 2% hematocrit followed by treatment with 2-BMP, ST092793, and 2-BMP+ST092793. Untreated parasites were taken as control. Following treatment, schizonts were incubated for 14 h. After 14 h, Giemsa-stained smears were made from treated and untreated parasites. The number of infected RBCs at the ring stage were counted in a total pool of 3,000 erythrocytes using a light microscope under ×100 oil immersion to calculate percent invasion, and data were plotted graphically.

### Checkerboard assay

Checkerboard assays were used to evaluate the effects of the combination of 2-BMP (palmitoylation inhibitor) and ST092793 (kinase inhibitor) against the malaria parasite (Mungthin et al., 1998; Semenov et al., 1998; Yapi et al., 2000). For this, schizont-stage parasites (42–44 h) were Percoll purified and dispensed in a 96-well plate at 2% hematocrit and 1% parasitemia. 2-BMP was added vertically at different concentration ranges while ST092793 was added horizontally at different drug concentration ranges in 8*8 format. As a result, the checkerboard consists of columns and rows in which each of the well along the x-axis contains drug 2-BMP at different concentrations (0.625, 1.25, 2.5, 5.0, 10.0, 20.0, and 40.0 µM) and that along the y-axis contains ST092793 at different concentrations (0.625, 1.25, 2.5, 5.0, 10.0, 20.0, and 40.0 µM) (King and Krogstad, 1983). The plate was incubated at 37°C in a humidified chamber for 14 h. The fractional inhibitory concentration (FIC index = FIC A + FIC B, where FIC A is the IC50 of drug (A) in combination/IC50 of drug A alone, and FIC B is the IC50 of drug B in combination/IC50 of drug B alone) of each drug was calculated and plotted as an isobologram. A straight diagonal line with an FIC index equal to 1 indicates an additive effect between drug A and drug B, a concave graph below the diagonal with an FIC index of less than 1 indicates a synergistic effect, and a convex curve above the diagonal with an FIC index of more than 0 indicates antagonism (Fivelman et al., 2004; Akoachere et al., 2005; Kelly et al., 2007).

### Ethics statement

Animal studies were performed following CPCSEA guidelines and approved by the Institutional Animal Ethics Committee (IEAC) of JNU. Female BALB/c mice were obtained from the Central Laboratory Animal Resources, JNU, New Delhi, and maintained under standard conditions. For experiments, donor blood was obtained from Rotary Blood Bank (RBB), New Delhi, India.

## Results

### Annotation of dually modified proteins in *Plasmodium falciparum*

**Figure 2:**
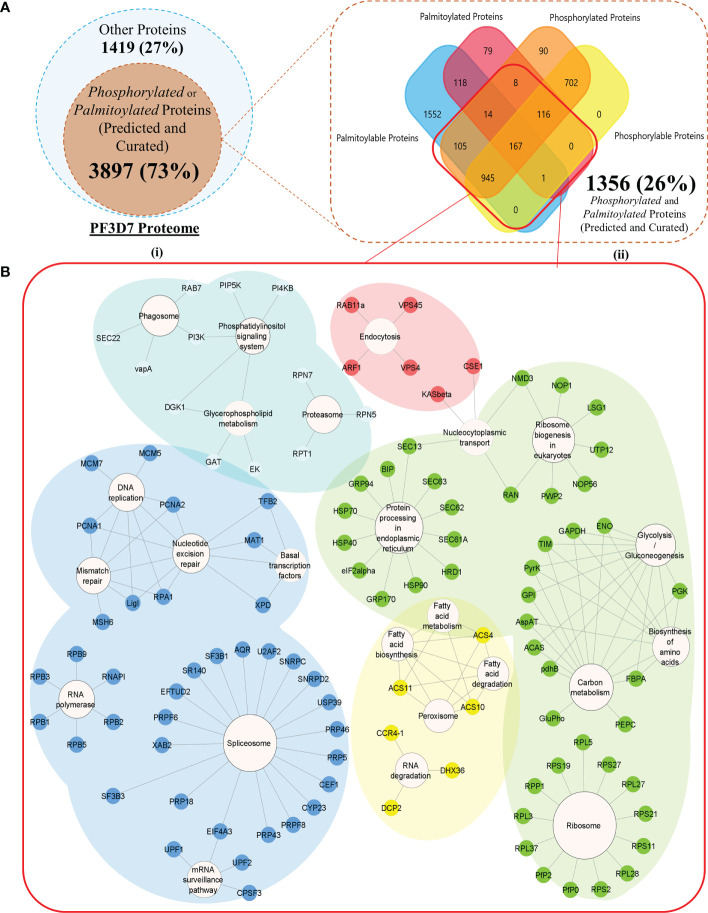
**(A)** (I) A Venn diagram representing all the *Modifiable* proteins (inner subset; dark orange) in the proteome of *Plasmodium falciparum* 3D7 (outer subset; light blue). (II) A Venn diagram with a more detailed breakdown of the proteins processed or predicted to be processed by palmitoylation and phosphorylation (the red box encapsulates all the dually modifiable proteins) **(B)** Biological processes enriched by proteins grouped into each of the five k-means clusters of the dually modifiable proteins. (Cluster 1 represented by Red, cluster 2 represented by Yellow, cluster 3 represented by Green, cluster 4 represented by Cyan, and cluster 5 represented by Blue nodes).

A total of 5,316 proteins, encoded by 5,387 gene transcripts, could be identified as components of the *P. falciparum* 3D7 proteome (**Supplementary Table 1**). PTM types such as palmitoylation and phosphorylation modulate *P. falciparum* proteins to critically resolve molecular processes responsible for parasite propagation and survival during the intraerythrocytic developmental stages. To understand the interdependence between PTMs and its effect on the parasite life cycle, we began by annotating a pool of proteins within the *P. falciparum* 3D7 proteome, which are susceptible to either phosphorylation or palmitoylation. A cumulative of 2,148 “high confidence” phosphoproteins could be compounded from curated sources (Ganter et al., 2017; Gupta et al., 2022), systematic search results (search term = “phosphoprotein” in UniProtKB, NCBI, and PlasmoDB), and Pf-Phospho prediction results (**Supplementary Table 2**). Phosphoproteins predicted by Pf-Phospho were labeled “Phosphorylable,” and those retrieved from curated resources and databases, using systematic searches, were labeled as “Phosphorylated.” In addition, 3,105 proteins were either predicted (Ren et al., 2008) or found in curated datasets (Jones et al., 2012)/databases (NCBI, UniProtKB, and PlasmoDB) to be components of the parasite palmitome (**Supplementary Table 3**). Palmitoylated proteins predicted by the CSS-Palm were termed “Palmitoylable,” and those retrieved from curated resources and databases, using systematic searches, were categorized as “Palmitoylated” proteins (**Figure 2A**).

A cumulative pool of 176,872 (non-redundant) amino acid residues were found as plausible modification sites in (3,762 isoforms of) 3,721 proteins (**Supplementary Table 4**). For the rest of the modifiable proteins (176), no site-specific information could be gathered. Cysteines are known to be preferred amino acid residues/sites for palmitoylation. However, serines (55%) were concluded to be the most phosphorylated amino acid residues/sites in *Plasmodium falciparum* 3D7 (**Supplementary Figure 1**).

All the predicted or curated PTM sites aligned into 44 and 210 significant palmitoylation and phosphorylation sequence windows or motifs, respectively (FDR much less than 1%). The sequence windows were discovered and ranked based on their overall scores using the MoMo tool (Cheng et al., 2019) of the MEME Suite (Bailey et al., 2015) (**Supplementary Table 5**).

Among all the modifiable or modified proteins, 1,356 proteins were annotated to be dually modifiable by curated resources or prediction results (**Supplementary Table 6**). A list of 1,327 proteins (97.86% out of all dually modifiable) could be mapped to the STRING database for functional and PPI annotations. To find the functional relevance of dual modification in the Plasmodium parasite, all the nodes were grouped into five different STRING-generated k-mean clusters using default settings (**Supplementary Table 7**). Cluster 4 (cyan) had a significant enrichment (p value below 1%) of the *P. falciparum* 3D7 glideosome motor machinery and invasion complex-related proteins retrieved from the PlasmoDB searches (filtered for only text-mined proteins and proteins studied in rodent malaria models). Cluster 4 was found to be significantly (q value below 5%) associated with critical parasite processes like phagocytosis, phosphatidylinositol signaling, glycerophospholipid metabolism, and proteasome abundance (**Figure 2B**). Each of the processes have time and again been reported to be guiding the intraerythrocytic development (IED) and plasticity of Plasmodium in dynamic hosts and microenvironmental conditions (Déchamps et al., 2010; Tawk et al., 2010; Krishnan and Williamson, 2018). The cyan cluster can be regarded as one of the most critical functional units of parasite motility that are (plausibly) guided by both phosphorylation and/or palmitoylation (**Supplementary Table 7**).

**Figure 3:**
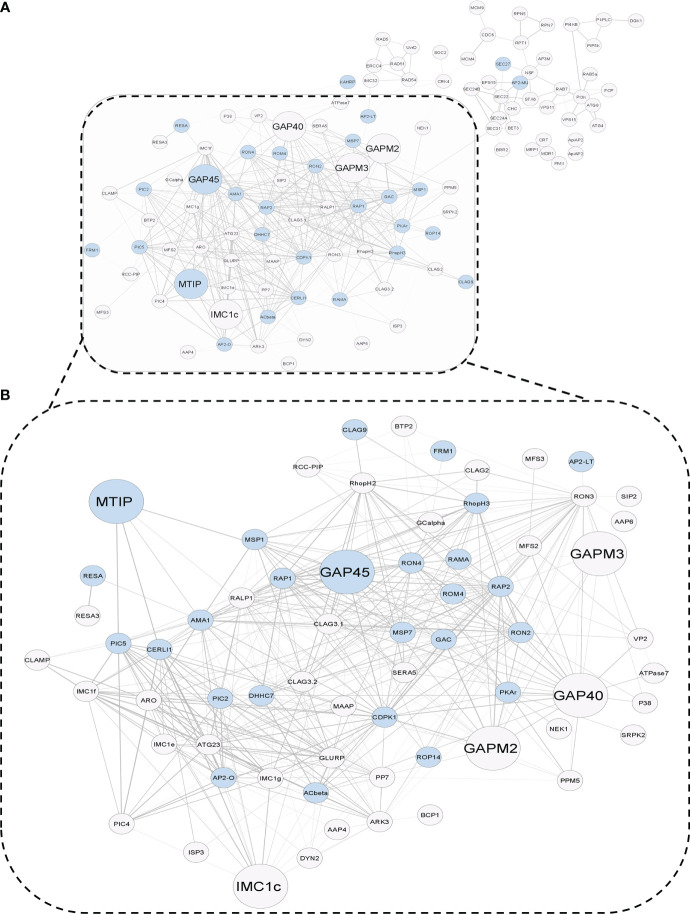
**(A)** STRING-generated Protein–Protein Interaction graph of dually modifiable proteins in k-means cluster 4. The PPI map draws a relationship between the molecular factors of parasitic invasion (blue nodes) and glideosome-mediated motility (larger nodes) in *P. falciparum* 3D7, which are processed by a crosstalk of *Palmitoylation* and *Phosphorylation*. **(B)** A functional subnetwork of major glideosome and invasion motor complex-related proteins, which has been termed as the infection-associated Motility and Invasion Complex (MIC) (dotted box).

Two hundred twenty-seven protein nodes, out of the 273 STRING-mapped and k-mean clustered protein nodes, were identified to have at least one edge connecting them to a certain other node in a PPI map created with the highest confidence score (0.9) and a high FDR stringency (below 1%). The basic PPI map generated by the STRING database was visualized using the Cytoscape application. Nodes without a protein name (or N/A as node label) (found for 105 proteins) and other disconnected nodes (10 proteins) were hidden to boost the simplicity of the graph (**Figure 3A**). A subnetwork with 66 nodes was found to be highly enriched (p value below 1%) with the key components of the actomyosin, and glideosome motor machineries, including GAP45, MTIP, CDPK1, and the essential light chains (ELCs), were renamed to “Motility and Invasion Complex” (MIC). These results indicated that the dual modification of MIC factors might be critical in the regulation of the parasitic motor complex or assembly responsible for the generation of a concerted force for the active invasion of the host cells. Within the MIC subnetwork, two core glideosome motor complex proteins, GAP45 and MTIP, were found to be the branching factors orchestrating both motility and general host-invasion processes in *P. falciparum* (**Figure 3B**).

### *In-silico* motif analysis revealed PTM crosstalk hotspots possibly regulating parasite motility and invasion

**Figure 4:**
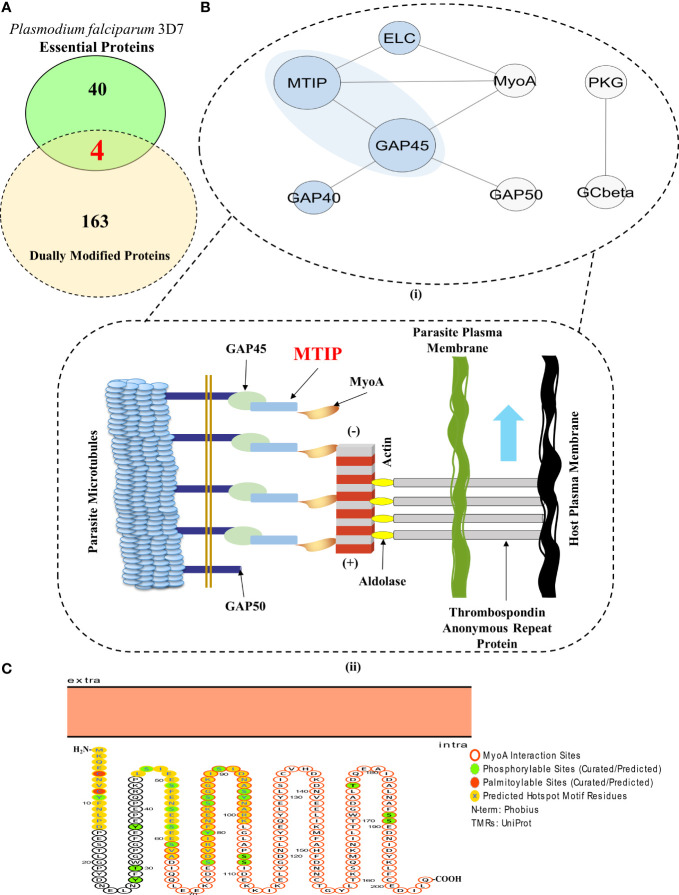
MTIP is an essential merozoite factor guiding parasite motility and erythrocytic invasion in hosts. **(A)** A Venn diagram showcasing the proteins which are dually modified by palmitoylation and phosphorylation and are essential for parasite survival. (Red fonts within the union of yellow and green ellipses; includes plasmepsin II, adenosine deaminase, calcium-dependent protein kinase 1 and myosin A-tail interacting protein). **(B)** (I) A STRING-generated PPI network of all the physical interactors constituting the glideosome motor complex. (Blue background) (II) An illustration of the actomyosin motor complex with MTIP as one of the central physical interactors. Revisualized from the published concepts of the glideosome motor unit (Bosch et al., 2006, Saunders et al., 2020). **(C)** A Protter generated illustration of MTIP showing the colocalization of phosphorylation and palmitoylation sites in the hotspots and MyoA interaction motif (Omasits et al., 2014, Anam et al., 2020).

Within a set of 1,356 proteins qualifying as dually modifiable, a common set between the prediction results and curated datasets/databases unveiled 167 proteins exhibiting propensities for both palmitoylation and phosphorylation (**Figure 1** and **Supplementary Table 6**). These 167 dually modified proteins were screened for essentiality using existing literature and systematic database searches (Aurrecoechea et al., 2009; Ali et al., 2021; Bateman et al., 2021). Based on this, four dually modified proteins, namely, PMII (plasmepsin II), ADA (adenosine deaminase), CDPK1, and MTIP, were found to be essential for parasite IED and invasion having no paralogs in human hosts (Ali et al., 2021) (**Figure 4A** and **Supplementary Table 6**). These four proteins were surveyed for the modification sites and discovery of the hotspot motifs, yielding a list of 100 unique modification sites for palmitoylation and phosphorylation. Non-redundant modification sites upon the four proteins could be classified into 10 elaborate hotspots. Motifs featuring amino acid residues susceptible to both palmitoylation and phosphorylation can be considered as the best probable grounds of complementary crosstalk between the PTM types. Only two (out of 10 PTM hotspots) could be identified as amino acid motifs with high propensity for both palmitoylation and phosphorylation. These motifs in the corresponding proteins CDPK1 and MTIP were thus termed as “Dually Modified” hotspots for complementary crosstalk (**Supplementary Table 8**), and both of these proteins could be detected in the MIC subnetwork (**Figure 3**). MTIP, however, features a lower frequency of polymorphisms across all strains in comparison to CDPK1. Also, MTIP is more conserved than CDPK1 within the Apicomplexa phylum and shows a higher proteomic expression during the merozoite, ring, and schizont stages of parasite infection (Szklarczyk et al., 2021; Amos et al., 2022). Interestingly, only MTIP serves as a core physical regulator of the actomyosin machinery, which is a critical functional unit that assists the glideosomes by inducing the potentiation of parasite movement and subsequent invasion processes in preferred hosts (**Figure 4B**). In addition, MTIP projects conserved amino acid motifs showcasing high propensity for PTMs and interactions with other proteins of functional significance like MyoA. All the three hotspots predicted in MTIP, including the dually modified hotspot region, are located within the window of the first 100 amino acids. The second hotspot region in MTIP features as many as five known phosphorylation sites and completely spans the disordered region of the protein (UniProtKB annotations). This asserts evolutionary significance for the predicted hotspot motif (**Figure 4C**).

The adapter protein, MTIP, mediates the formation of the MyoA–MTIP complex that associates with co-expressing cytosolic GAP45 and relocates to the developing IMC, predominantly during early schizogony when the merozoites are released [**Figure 4B (II)**]. CytoHubba application-based Multiple Clique Centrality (MCC) scoring revealed the MyoA-MTIP-GAP45 (shared rank 1, MCC score = 4.0) complex as the core hub of the physical protein association networks underlying the glideosome motor complex (**Supplementary Table 9**). Since MTIP is a mediator of the MyoA : MTIP-GAP45 complex formation, it is critical to the integrity of the motility and erythrocytic invasion complex in the parasites (Bosch et al., 2006; Ridzuan et al., 2012). Although the expression patterns of MTIP are not specific to any particular growth stages, the protein or the corresponding mRNAs are highly expressed in the merozoites (both short and long lived) and the schizonts (Pease et al., 2013; Kumar et al., 2017a). Additionally, the functional implications of its protein orthologs in *P. berghei* establishes MTIP among those adapter protein-coding genes which are extremely essential for growth and propagation during the asexual stages of the Plasmodium life cycle (Bushell et al., 2017; Howick et al., 2019).

An extensive survey of proteins and PTMs depicts the presence of distinct and coinciding phosphorylation and palmitoylation motifs in crucial factors like PMII, CDPK1, and MTIP that govern *P. falciparum* infection. PTM events and their hotspots could be located in many proteins involved in discriminatory aspects of infection by Plasmodium spp. parasites. Thus, the cross-talking PTM events and hotspots, beyond proteins and mRNAs, can be explored as key molecular targets to expand the druggability of the critical Plasmodium spp. factors like the protein components of the MyoA : MTIP motor complex. Subsequently, MTIP was concluded as one of the most essential factors for asexual stage growth of parasites which featured multiple hotspot stretches with a concentrated chance of PTM crosstalk events between palmitoylation and phosphorylation.

### The dynamic interplay between phosphorylation and palmitoylation status of MTIP

Individual studies have demonstrated that the *P. falciparum* protein machinery of invasion gets phosphorylated and palmitoylated (Rees-Channer et al., 2006; Alam et al., 2015; Edmonds et al., 2017; Schlott et al., 2018). Blocking these PTMs in glideosome-associated proteins leads to invasion inhibition (Yakubu et al., 2018). MTIP is known to be phosphorylated at serine residues 47, 51, 55, 58, 61, 107, and 108 (Green et al., 2008; Douse et al., 2012) and palmitoylated at cysteine 5 and 8 amino acid residues (Jones et al., 2012).

**Figure 5:**
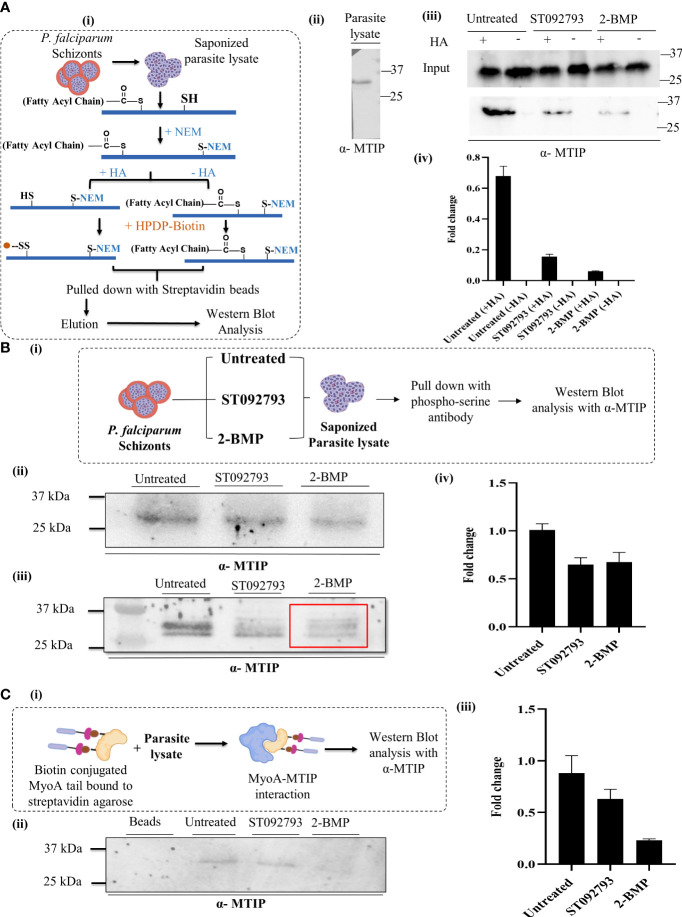
**(A)** (I) A schematic representation of acyl biotin exchange analysis to detect palmitoylation of parasite protein coupled with immunoblot analysis. (II) *P. falciparum* 3D7 lysate blotted using mouse anti-MTIP antibody (1:20,000 dilution in phosphate-buffered saline 0.1% Tween 20). A single band at 24 kDa representing MTIP was observed. (III) The representative immunoblot displayed inputs (upper panel) representing MTIP expression levels in untreated lysates and lysates treated with ST092793 and 2-BMP in presence and absence of HA. Immunoblots represent the MTIP expression in the ABE pulled fraction with and without HA treatment (lower panel). Low intensity of MTIP expression was detected in ST092793 and 2-BMP treated ABE pulled-down fraction in comparison to control. (IV) Bar graph represents the fold change in MTIP band intensity before and after ABE pull down. Two independent experiments have been performed, n = 2. **(B)** (I) Schematic representation to analyze the dynamic interplay between phosphorylation and palmitoylation status of MTIP. Schizont stage parasite were treated with ST092793, BMP alone. After treatment parasite lysates were subjected to pull-down analysis using phospho-serine antibody, eluate fractions were then probed with anti-MTIP antibodies. (II) Immunoblot represents the input showing equal MTIP expression in untreated and ST092793- and 2-BMP treated samples. (III) In the presence of 50 µM 2-BMP, the phosphorylation of MTIP (measured by pull-down using phospho-Ser antibody followed by probing with anti-MTIP) was predominantly reduced (red box) as compared to MTIP in untreated lane. (IV) Bar graph represents the change in fold intensity before and after pull-down using phospho-Ser antibody. **(C)** (I) Schematic representation of workflow to analyze the MTIP interaction with MyoA tail. (II, III) The immunoblot showed the synergistic impact of dual PTM on MTIP crosstalk with myosin A tail, in the presence of 50 µM 2-BMP. There was no MTIP band following pull-down using biotinylated myosin A tail as the bait. The graph denotes fold change in intensity of MTIP in comparison to inputs taken before pull-down with myosin A tail in individual lanes. Two independent experiments have been performed.

To answer whether silencing of one PTM type in MTIP would affect the other modification types, we used two PTM-specific inhibitors: (i) 2-bromopalmitate (2-BMP), a generic inhibitor of palmitoyl acyltransferases (Resh, 2006) that has been shown to block palmitoylation in *P. falciparum* (Jones et al., 2012), and (ii) ST092793 (Jain et al., 2020), a novel broad-spectrum phosphorylation inhibitor shown to have strong inhibitory ability against pan-kinases during the intra-erythrocytic development of parasites, as shown by a previous study from our lab (Jain et al., 2020). A brief schematic of the ABE protocols is provided in **Figure 5A (I)**. MTIP in parasite lysates was detected using an anti-MTIP antibody that gave a single band at 24 kDa after probing with *P. falciparum* lysates [**Figure 5A (II)**]. To ensure if the reduction in MTIP levels was due to crosstalk, but not because of the treatments, the levels of MTIP in input lysate after ST092793 and 2-BMP treatments in the presence and absence of hydroxylamine treatment were checked, which indicated no change in MTIP band intensities [**Figure 5A (III)**]. The palmitoylation status of MTIP was evaluated in the ABE-enriched purified palmitome (Wan et al., 2007; Edmonds et al., 2017) after ST092793 and 2-BMP treatments. The results demonstrated reduced band intensity of MTIP indicating attenuated palmitoylation of MTIP upon phosphorylation inhibition [**Figure 5A (III, IV)**], whereas a further reduced MTIP band was detected in 2-BMP (+HA wells) treatment in comparison to ST092793 confirming successful inhibition of palmitoylation that was used as a positive control [**Figure 5A (III)**]. The same was depicted in an intensity plot represented as bar graph [**Figure 5A (IV)**], which represents the fold change in intensity before and after ABE pull-down by streptavidin beads.

To understand whether the phosphorylation status of MTIP might also be regulated by palmitoylation, we first enriched the phosphorylated pool of *P. falciparum* using a phosphoserine-specific antibody from 2-BMP-treated lysate and identified the phosphorylation status of MTIP in the same. A brief description of the methodologies is provided in **Figure 5B (I)**. To ensure the equal level of MTIP in all the samples, input lysates after ST092793 and 2-BMP were checked, which indicated no change in MTIP band intensities [**Figure 5B (II)**]. This was considered as a control/input. Probing with phospho-serine antibodies showed decreased intensity of phosphorylated MTIP suggesting palmitoylation-dependent phosphorylation [**Figure 5B (III)**]. Additionally, densitometry analysis was performed for the pull-down assays and represented as fold change in MTIP intensity after pull-down analysis [**Figure 5B (IV)**].

### Effect of PTM crosstalk on MTIP’s interaction with its primary motor complex partner Myosin A

The myosin A/myosin A tail interacting protein (MyoA-MTIP) complex is a notable molecular bridge of motor machinery that is modified post protein translation to drive the parasite entry into human RBCs (Bosch et al., 2006; Green et al., 2006). Precisely, the phospho motifs lying in the C-terminal domain of MTIP interact with only the tail constituting 798–818 amino acids of myosin A (Bergman et al., 2003; Baum et al., 2006; Bosch et al., 2006; Green et al., 2006; Thomas et al., 2010; Khamrui et al., 2013). However, it is unknown whether blocking the palmitoylation-phosphorylation crosstalk has any impact on the myosin A/myosin A tail interacting protein (MyoA-MTIP) complex. To answer this, we used a synthetic biotinylated peptide mimicking the tail domain of myosin A that is sufficient for detecting its interaction to MTIP. This peptide was used as bait to pull down MTIP from the lysate treated with both phosphorylation and palmitoylation inhibitors sequentially [**Figure 5C (I)**]. The untreated lysate was used as a positive control. The data suggested that inhibiting palmitoylation by 2-BMP led to an abolished interaction of the MTIP C-terminal domain with the myosin A tail, while blocking phosphorylation by ST092793 demonstrated a diminished interaction [**Figure 5C (II)**] as shown by the reduced intensity of MTIP [**Figure 5C (III)**]. Findings from the study suggested that there might be a strong possibility of PTM crosstalk on amino acid domains of MTIP interacting with the myosin A tail.

### Global crosstalk between phosphorylation and palmitoylation in *P. falciparum*

**Figure 6:**
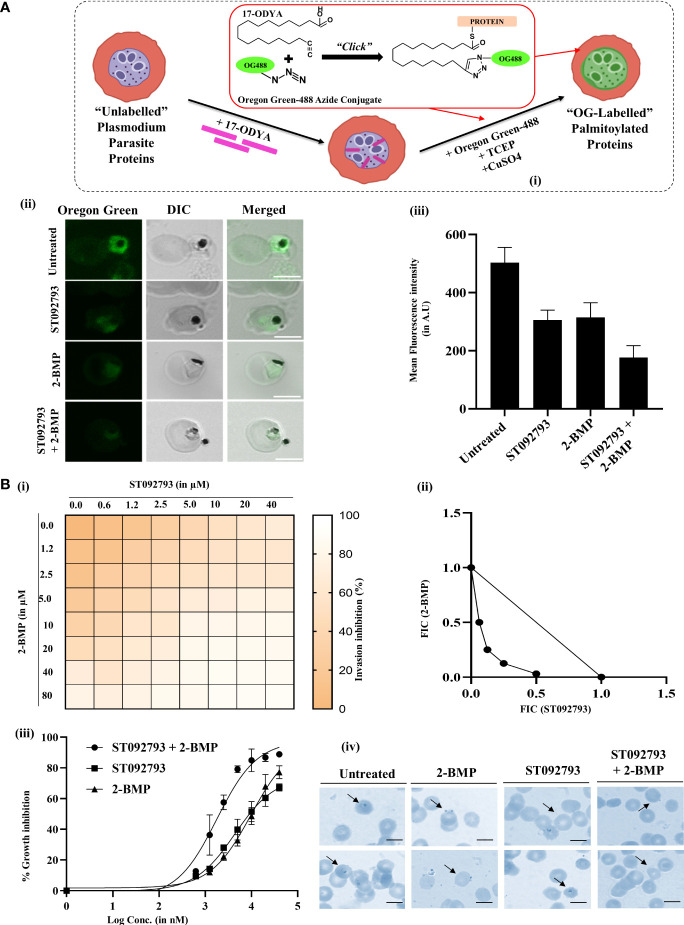
**(A)** (I) Schematic of the click chemistry approach for imaging *in situ* protein palmitoylation in malaria parasite during asexual development. Intraerythrocytic parasites were metabolically labeled with 17-ODYA (palmitic-acid analogue) followed by labeling with Oregon Green 488 in the presence of TCEP and copper sulfate. (II) Clickable metabolic labeling of the *P. falciparum* parasites following ST092793, 2-BMP, and ST092793+2-BMP treatment. The weak palmitoylation profiles in comparison to untreated parasites, especially in the case of ST092793, were observed. Scale bar indicates 5 µm. (III) Bar graphs represent the mean fluorescence intensity (MFI) and denote the palmitoylation profile in treated and untreated erythrocytes, where 20 cells were used for calculation for two biological replicates. **(B)** (I) The heat plot of invasion inhibition in 3D7 in the presence of ST092793 and 2-BMP when present alone and in combination (ST092793 + 2-BMP). ST092793 was added horizontally in 96-well plates (0, 0.6, 1.2, 2.5, 5.0, 10, 20, 40 µM) while 2-BMP was added vertically (0, 0.6, 1.2, 2.5, 5.0, 10, 20, 40, 80 µM) in 8*8 format. Dose–response matrices from 0% to 100% indicate different percentages of invasion inhibition. (II) The isobologram analysis of 2-BMP and ST092793 which shows a synergistic effect when used in combination against the 3D7 strain of *P. falciparum* with FIC index of <1. (III) The dose–response curve for 2-BMP and ST092793 when used alone and in combination. (IV) Giemsa-stained smears from 2-BMP-, ST092793-, and 2BMP+ST092793-treated parasites. Arrowhead indicates the ring formation after successful invasion, while invasion defect was observed in the case of 2-BMP-, ST092793-, and 2BMP + ST092793-treated parasites.

Click chemistry is a direct and well-defined tool to evaluate global palmitoylation (Jones et al., 2012; Ayana et al., 2018; Yadav et al., 2019) where all the probable palmitoyl motif-containing groups are labeled with Oregon Green 488 dye. By using click chemistry, the palmitome of parasites could be visualized [**Figure 6A (I)**]. The perturbations in the global palmitome of *P. falciparum* due to phosphorylation inhibition were assessed by using this assay, following treatment with ST092793. For this, *P. falciparum* parasites were treated with ST092793 (10 µM), 2-BMP (50 µM), and ST092793+2-BMP (10 µM, 50 µM respectively) in combination followed by visualization of palmitoylated proteins by measuring the fluorescence of incorporated palmitic acid analogue Oregon Green 488 dye as shown represented in the respective intensity plot [**Figure 6 A (II, III)**]. A 50% reduction in fluorescence intensity of ST092793-treated parasites as compared to control showed the effect of phosphorylation inhibition on global *P. falciparum* palmitome [**Figure 6A (III)**], thereby confirming the crosstalk between phosphorylation and palmitoylation at a global level in *P. falciparum*. A decrease in fluorescence intensity in 2-BMP-treated parasites confirmed effective palmitoylation blockage. Additionally, the impact of palmitoylation inhibition was much more evident in the case of ST092793+2-BMP [**Figure 6A (III)**]. Overall, these results confirmed crosstalk between phosphorylation and palmitoylation in the *P. falciparum* proteome.

### Phosphorylation and palmitoylation show synergistic effects in *P. falciparum* invasion

Accumulating evidence has shown that global phosphorylation of *P. falciparum* proteome governs many cellular processes including invasion (Ekka et al., 2020; More et al., 2020; Perrin et al., 2020). In addition, there are scanty findings available that also suggest that global palmitoylation of glideosome-actinomycin motor assembly proteins in *P. falciparum* might impact the invasion phenotypes (Jones et al., 2012). However, there is a lacuna in the understanding of whether both these modifications might need to act synergistically during the invasion.

To explore this hypothesis, we have studied the global synergism of these dual PTMs on *P. falciparum* invasion using palmitoylation and phosphorylation inhibitors 2-BMP and ST092793, respectively. To check whether these two drugs, 2-BMP (palmitoylation inhibitor) and ST092793 (kinase inhibitor), when used in combination against malaria parasite show a synergistic effect, the *in vitro* isobologram method was used (Fivelman et al., 2004; Akoachere et al., 2005; Kelly et al., 2007). For this, schizont-stage parasites (42–44 h) were treated with each of the drugs alone and in combination at different concentrations. The Giemsa smears were made after 14 h of incubation, and parasitemia was calculated. The FIC index was calculated for each drug concentration and plotted as an isobologram. The result indicated that 2-BMP and ST092793 when used in combination show a synergistic effect *in vitro* with an FIC index of less than < 1 [**Figure 6B (I, II)**]. Also, a dose–response curve was plotted for each of the drug alone and in combination and the result showed that when the parasite was treated alone with each drug alone at different concentrations IC50 came out to be 20 and 10 µM, respectively, while when used in different drug doses in combination, IC50 shifts to 2 µM [**Figure 6B (III)**], indicating a synergistic effect of palmitoylation and phosphorylation when inhibited together *via* 2-BMP and ST092793, respectively.

## Discussion

The integrated proteomic analysis provides new insights into PTMs, the biological building blocks underlying the functional diversity of proteins in eukaryotic organisms (Yakubu et al., 2018; Andrés et al., 2020). Emerging studies have shown the implications of the crosstalk of PTMs and their diverse roles across the spectrum of diseases (Hunter, 2007; Yakubu et al., 2018; Habibian and Ferguson, 2019). Accumulating evidence has shown that multiple PTMs like phosphorylation, palmitoylation, glycosylation, acetylation, ubiquitylation, and myristoylation are abundant in Apicomplexan parasites (Douse et al., 2012; Yakubu et al., 2018). These PTMs govern basic steps like motility, host–parasite interaction, cellular homeostasis, and infectivity (Chuenkova et al., 2001; Soulat and Bogdan, 2017; Damianou et al., 2020; Ekka et al., 2020; Perrin et al., 2020).

Existing data also suggest that in the case of *P. falciparum*, PTMs regulate malaria disease progression (Hunter, 2007; Lonard and O’Malley, 2007; Yao et al., 2011; Swaney et al., 2013). Thus, the PTM sites and crosstalk among multiple biologically implicated PTM types can be targeted in Apicomplexan parasites which has a pronounced effect in overall parasite growth and host invasion. Phosphoproteome and palmitome analyses of *P. falciparum* have revealed multiple roles of these PTMs in invasion, survival, and progression (Treeck et al., 2011; Jones et al., 2012; Alam et al., 2015; Hodson et al., 2015; Lasonder et al., 2015). In other eukaryotic systems, the crosstalk between palmitoylation and phosphorylation is well-defined and has been correlated with various cellular functions (Hunter, 2007; Yao et al., 2011; Ahner et al., 2013; Vu et al., 2018) also linked to cardiovascular diseases (Habibian and Ferguson, 2019; Aggarwal et al., 2020). In *Plasmodium*, phosphorylation and palmitoylation are the abundant kinds of PTMs (Treeck et al., 2011; Jones et al., 2012; Lasonder et al., 2015; Park et al., 2015). Hence, it makes sense for the parasite to use these in order to fine-tune the cellular events for biological homeostasis. Thus, deciphering the crosstalk between phosphorylation and palmitoylation and the key regulatory hub proteins will aid in understanding the parasite’s response mechanisms during parasite life-cycle progression and invasion.

To bridge this gap in understanding, we have tried to address a fundamental question, if phosphorylation and palmitoylation in *P. falciparum* act in dynamic interplay and are interdependent. In the direction, we introduced a new strategy involving de-convolution of (i) the cumulative catalog of PTM partners for phosphorylation and palmitoylation, (ii) common candidate proteins showcasing the dual PTMs, and (iii) dually modified regulatory hub proteins and their interacting partners that are committed to Plasmodium invasion. Moving ahead, first, we studied the global crosstalk between the phosphoproteome and palmitome of *P. falciparum* by *in silico* analysis that enriched the repertoire of proteins and underlying motifs and hotspots with dual PTM types. Although the predicted phosphorylation and palmitoylation hotspot motifs on these *Plasmodium* proteins may get exposed during different stages of the asexual life cycle of the parasite, our click chemistry analysis proved that crosstalk exists between phosphorylation and palmitoylation at the global proteome level during RBC infection stages of the parasite. The phenotypic effect of crosstalk was further studied by measuring *P. falciparum* growth inhibition against individual and combinatorial treatments with ST092793 and 2-BMP which are generic inhibitors of phosphorylation and palmitoylation, respectively. Although 2-BMP is known to have non-specific effects (DeJesus and Bizzozero, 2002; Zheng et al., 2015), it has been used extensively to target palmitoylation-based pathways in *L. donovani*, *T. cruzi*, and *Toxoplasma* (Foe et al., 2015; Ayana et al., 2018; Batista et al., 2018b; Batista et al., 2018a) for reducing infectivity. Plausibly, one of our recent unpublished data has also shown that 2-BMP has no impact on overall morphology of host RBCs. Interestingly, some recent studies have identified certain erythrocytic kinase specific inhibitors targeting phosphorylation, with promising antimalarial activity (Kesely et al., 2016; Pantaleo et al., 2017). A recent published study from our laboratory has also detected ST092793 as the novel kinase inhibitor from the MyriaScreen II diversity collection library with promising antimalarial activity (Jain et al., 2020). Our data suggested that simultaneous treatment with ST092793 and 2-BMP, targeting phosphorylation and palmitoylation, respectively, in *P. falciparum* imposed strong synergistic effects on parasite growth inhibition, suggesting a significant impact of these modification types in shaping the intracellular growth dynamics of *P. falciparum*.

Next, we aimed to identify the crucial regulatory hub proteins and their interacting partners among the enriched repertoire of dually modified proteins responsible for parasite invasion processes. Merozoite invasion is a sequential process involving initial attachment of the merozoite to RBC, tight attachment, tight junction formation, and penetration involving multiple machinery proteins including myosin A, MTIP, GAP45, GAP50, ELC, and TRAP (Baum et al., 2006; Jones et al., 2006; Frénal et al., 2010; Thomas et al., 2010). In our analysis, we found MTIP as the common emerging and hub protein which is also a key protein in the motility and invasion assembly controlling invasion. The role of the glideosome motor complex of which MTIP is a main member is well established by previous studies (Green et al., 2006; Turley et al., 2013). We asked if the dual PTM crosstalk might play any decisive role in underlying MTIP expression and its interaction with its target proteins during pre-invasion and invasion processes. Our *in vitro* data suggested that the regulation machineries of palmitoylation and phosphorylation of MTIP are strongly interdependent.

Next, we identified the interacting partners of the glideosome motor complex in *P. falciparum* that are modulated synergistically through phosphorylation and palmitoylation. Among these partners, we found the myosin A-MTIP complex to be strictly regulated by the dual PTM modifications of MTIP. It is also evident from our *in vitro* data that the molecular crosstalk between both phosphorylation and palmitoylation events in MTIP governs its possible interaction with myosin A (**Figure 6**). Based on these data, we suggest that dual PTM crosstalk in MTIP might have a role forming the myosin A–MTIP complex, critical for host invasion at the merozoite point of entry in the host erythrocytes.

Overall, our analysis has provided two important insights: (i) dual PTM crosstalk governs the MTIP-dependent molecular pathway involved in *P. falciparum* pre-invasion- and invasion-associated processes, and (ii) a combination of drugs targeting both palmitoylation and phosphorylation can provide a novel antimalarial therapeutic strategy that can offer the advantage of improved efficacy with reduced drug resistance.

## Conclusion

The datasets presented herein provide the first evidence of the crosstalk between palmitoylation and phosphorylation in *P. falciparum* motility and parasite-mediated cell invasion. PTM crosstalk events and motifs discussed in the article may be beneficial for the development of novel chemotherapeutics. The strategies devised for the study may further be applied to research the crosstalk possibilities among multiple other PTM types, and underlying cell-regulatory networks in other apicomplexan parasites. Additionally, individual datasets, catalogs, and findings from the study have been made available and may be referred to as an up-to-date repository of palmitoylation and phosphorylation modification events and hotspots in *Plasmodium falciparum* (isolate 3D7) proteins.

## Data availability statement

The original contributions presented in the study are included in the article/**Supplementary Material**. Further inquiries can be directed to the corresponding authors.

## Author contributions

Conceptualization, SS, AR, ZA. Methodology, SS, SP, AR. Experimental design and execution ZA, GK, DR, SM, NG, AK, AC, NS, SK, JS, AR, SS. Software and bioinformatics TP, DR, SR, SM, SP. Validation, AR, SS and ZA. Investigation, GK, ZA. Resources, AR, SS. Data compilation, ZA, SM, GK, SP, AR, SS. Writing—original draft preparation, ZA, SP, AR, SS. Writing—review and editing, SP, AR, SS, ZA, SM. Visualization and malaria culture GK, RS, SB, PM. Supervision, AR, SS. Project administration, AR, SS. Funding acquisition, AR, SS. All authors contributed to the article and approved the submitted version.

## Funding

This research was funded by the Department of Science and Technology, SERB grant number CRG/2019/00223 (AR, SS), and Drug and Pharmaceuticals Research Programme (DPRP, Project No. P/569/2016-1/TDT) (SS); the APC was funded by the Department of Science and Technology, SERB, IRHPA IPA/2020/000007 (AR, SS). SP is grateful for the funding support from the Cognitive Science Research Initiative (CSRI) program of the Department of Science and Technology (DST/CSRI/2018/247).

## Conflict of interest

The authors declare that the research was conducted in the absence of any commercial or financial relationships that could be construed as a potential conflict of interest.

## Publisher’s note

All claims expressed in this article are solely those of the authors and do not necessarily represent those of their affiliated organizations, or those of the publisher, the editors and the reviewers. Any product that may be evaluated in this article, or claim that may be made by its manufacturer, is not guaranteed or endorsed by the publisher.

## Supplementary material

Supplementary Figure 1
**(A)** A bar graph comparison of amino acids preferred for phosphorylation and palmitoylation modification types (cataloged for the underlying study from all sources). **(B)** Amino acids of preference for phosphorylation cataloged from only curated datasets (Dotted Red Box) suggest Serines (83%) and Tyrosines (3%) as the most and least phosphorylated residues in *Plasmodium falciparum* 3D7. **(C)** Absolute frequencies and percentages of amino acid residues preferred as phosphorylation and palmitoylation sites (from the total pool of PTM sites cataloged for the underlying study from all sources).

Supplementary Table 1 Total proteome of *Plasmodium falciparum* 3D7 cataloged for the study.

Supplementary Table 2 Cumulative phosphoproteome of *Plasmodium falciparum* 3D7.

Supplementary Table 3 Cumulative palmitome of *Plasmodium falciparum* 3D7.

Supplementary Table 4 A list of all the PTM events and sites pertaining to *Plasmodium falciparum* 3D7 proteome.

Supplementary Table 5 Consensus palmitoylation and phosphorylation sequence windows discovered using the MoMo web interface along with the sequence logo graphs.

Supplementary Table 6 A catalog of all the “Dually Modifiable” and “Dually Modified” *Plasmodium falciparum* 3D7 proteins.

Supplementary Table 7 The results generated from the k-means clustering of the dually modifiable *Plasmodium falciparum* 3D7 proteins.

Supplementary Table 8 PTM hotspot regions in all the modifiable proteins acquired from curated datasets/databases and prediction results, including PTM events and hotspots distribution on dually modified *Plasmodium falciparum* 3D7 proteins essential for its survival.

Supplementary Table 9 A tabular representation of the physical PPI map constituting the Glideosome motor complex of *Plasmodium falciparum* 3D7 as nodes for the Cytohubba based identification of top interactors.

## Abbreviations

MTIP, Myosin A tail interacting protein; PTM, Post-translational modifications; CDPKs, Ca^2+^-dependent protein kinases; GAPs, Glideosome-associated proteins; 2-BMP, 2-Bromopalmitate.
