# Supplementary figures and images for: Complementary crosstalk between palmitoylation and phosphorylation events in MTIP regulates its role during Plasmodium falciparum invasion
Source: Front Cell Infect Microbiol. 2022 Sep 29;12:924424. doi: 10.3389/fcimb.2022.924424 (PMC9556994; doi:10.3389/fcimb.2022.924424)

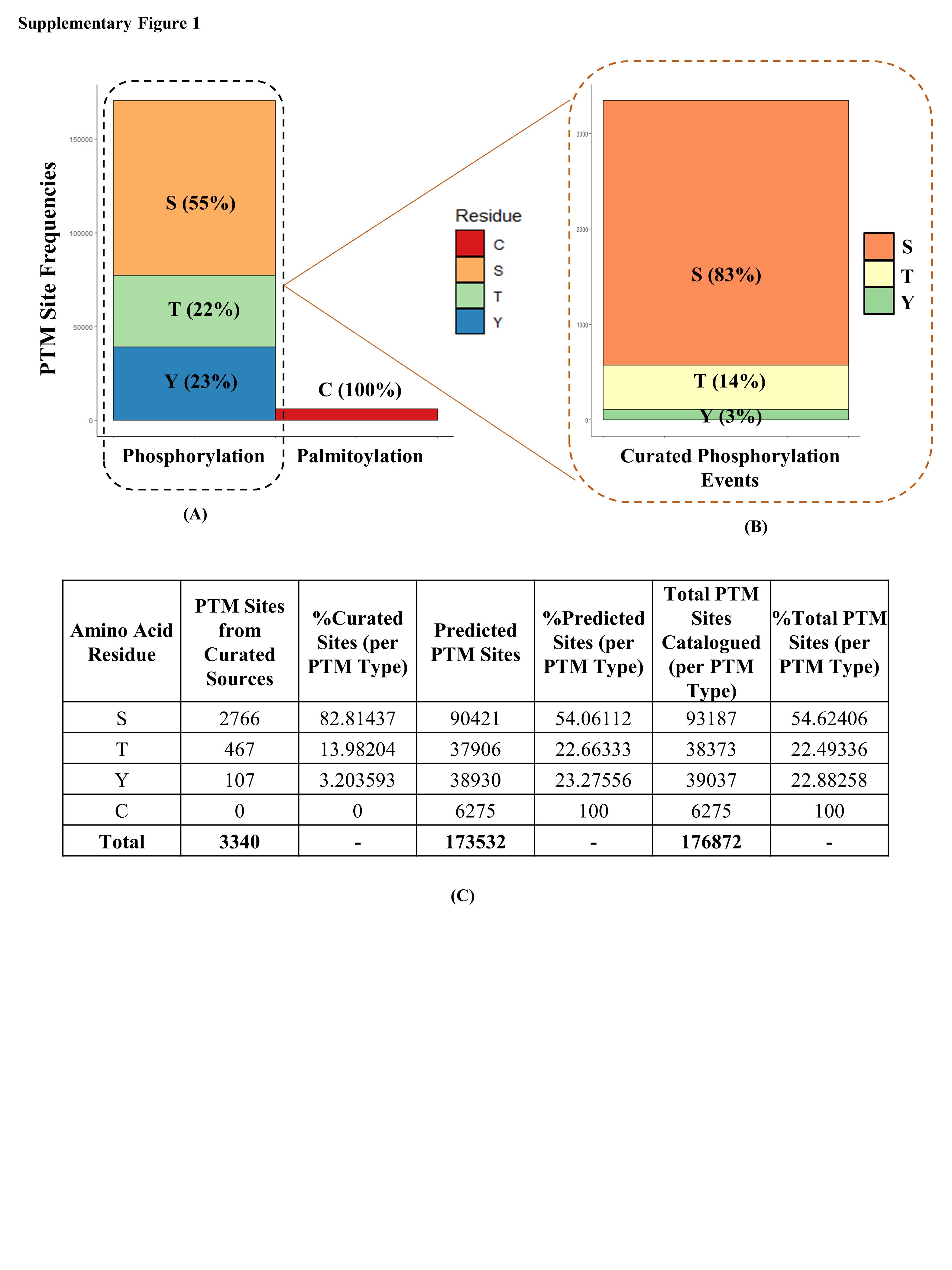

Supplement: Supplementary Figure 1 — (A) A bar graph comparison of amino acids preferred for phosphorylation and palmitoylation modification types (cataloged for the underlying study from all sources). (B) Amino acids of preference for phosphorylation cataloged from only curated datasets (Dotted Red Box) suggest Serines (83%) and Tyrosines (3%) as the most and least phosphorylated residues in Plasmodium falciparum 3D7. (C) Absolute frequencies and percentages of amino acid residues preferred as phosphorylation and palmitoylation sites (from the total pool of PTM sites cataloged for the underlying study from all sources). [file Image_1.tif]
